# Advances in the treatment of opioid use disorders

**DOI:** 10.12688/f1000research.10184.1

**Published:** 2017-01-27

**Authors:** George E. Woody

**Affiliations:** 1 Department of Psychiatry, Perelman School of Medicine at the University of Pennsylvania, 3440 Market Street, 3rd Floor, Philadelphia, USA

**Keywords:** naltrexone, buprenorphine, opioid use disorders, methadone, substance abuse, heroin

## Abstract

The development of medications for treating persons with opioid use disorders has expanded the number of evidence-based treatment options, particularly for persons with the most severe disorders. It has also improved outcomes compared to psychosocial treatment alone and expanded treatment availability by increasing the number of physicians involved in treatment and the settings where patients can be treated. The medications include methadone, buprenorphine, buprenorphine/naloxone, and extended-release injectable naltrexone. Studies have shown that they are most effective when used over an extended, but as-yet-unspecified, period of time and with counseling and other services, particularly for the many with psychosocial problems. Though controversial in some cultures, well-designed studies in Switzerland, the Netherlands, Germany, and Canada have demonstrated the efficacy of supervised heroin injecting for persons who responded poorly to other treatments, and this treatment option has been approved by Switzerland and a few other E.U. countries. The degree to which medication-assisted therapies are available is dependent on many variables, including national and local regulations, preferences of individual providers and their geographical location, treatment costs, and insurance policies. Greater availability of medication-assisted therapies has become a major focus in the U.S. and Canada, where there has been a marked increase in deaths associated with heroin and prescription opioid use. This paper provides a brief summary of these developments.

## Background

Opioid use disorders typically begin in the late teens or early twenties, occur at varying levels of severity, follow a course of remissions and relapses, and are associated with serious psychosocial and medical problems- including premature death due to overdoses, accidents, or substance-related medical problems (DSM-5, 2013). Sustained remission occurs in a significant minority of individuals, but it usually takes 10 or more years to emerge ^1^, and many survivors have medical and psychosocial problems that permanently impair their health, chances for employment, and overall adjustment.

Treatment does not cure these disorders, but it can change their course by reducing opioid use and its adverse effects. Among the first effective therapies were therapeutic communities (TCs). These emerged before medication-assisted therapies were available, and, though dropout rates are high, studies showed that over 80% of individuals who completed them had sustained remission and demonstrated significant improvement in overall adjustment ^2, 3^. The Dole and Nyswander methadone studies ^4^ introduced the first effective pharmacotherapy that is now used in many countries, where it is administered in the context of regulations that often limit its use to approved providers and mandate varying levels of observed dosing to reduce the chances for diversion and its associated adverse events.

Though methadone maintenance has become part of the landscape of opioid addiction treatment, it has not been universally accepted or integrated with general healthcare in some settings. For example, it is against the law in Russia and some former Soviet states, and approval for its use took over 30 years in some U.S. states. In the U.S. and some other countries (Republic of Georgia, for example), it is allowed only in specially licensed programs with funding streams and patient records that are separated from general healthcare and frequent inspections to check for compliance with regulations. It is not as highly regulated or isolated in some E.U. countries, Canada, or Australia, and some methadone programs (in Jakarta, for example) are sited in community health centers where medical services are readily available.

The last 20 years have seen studies showing that medically supervised heroin injection can be helpful for patients who have not responded to other therapies and the approval of two new medication therapies. The first of these two was buprenorphine, a partial agonist at the μ-opioid receptor that was discovered in the mid-1960s by John Lewis in the laboratories of Reckitt & Colman, a home products company based in the UK ^5^; the second was extended-release naltrexone.

## Buprenorphine

Buprenorphine is a schedule III, μ-opioid partial agonist with a greater margin of safety than full agonists and a less intense withdrawal ^5– 8^. It is approved in the U.S. for treating persons aged 16 years and older, although it has been studied mainly in adults who were addicted for 5 to 10 years or longer. It has been combined with naloxone in a 4:1 ratio to reduce abuse if crushed and injected, and a recent study found that this combination reduced its “street” value, often a surrogate for abuse liability.

Some countries regulate buprenorphine similarly to methadone, while others allow it to be used under less stringent conditions. For example, France allowed buprenorphine to be prescribed by general practitioners and dispensed in retail pharmacies throughout the country. This policy markedly increased the number of opioid-addicted individuals in treatment, reduced the number of heroin overdose deaths by four-fifths between 1994 and 2002, and reduced the prevalence of HIV infection among injecting drug users from 25% in the mid-1990s to 6% in 2010 ^9^.

The U.S has fewer regulations for buprenorphine than it does for methadone but more than France for buprenorphine. For example, the Drug Addiction Treatment Act (DATA) of 2000 ^10^ allowed physicians who have a waiver from the U.S. Substance Abuse and Mental Health Health Services Administration to prescribe Schedule III, IV, and V medications for persons with severe opioid use disorders and had an initial limit of 30 patients per physician. This limit was later increased to 100, and recent changes allow physicians who have prescribed buprenorphine to 100 patients for a year or more to apply for permission to increase their caseload to 275.

The passage of DATA 2000 was accompanied by studies showing that buprenorphine/naloxone is safe and effective when used in office-based practice ^11, 12^, can be effective without intensive counseling ^13^, can improve adherence to antiretroviral therapy and outcomes of patients being treated for HIV ^14, 15^, and is effective for opioid-addicted youth ^16, 17^ as well as persons addicted to prescription opioids ^18^. These developments increased the number of patients in opioid addiction treatment and the number of physicians treating them, and helped integrate opioid addiction treatment into general health care in the U.S.

However, these advances have not been without problems. The absence of strict regulations on observed dosing has been associated with significant buprenophine diversion. This problem has been a major concern in the U.S. but has been associated with less morbidity and mortality than with diversion of methadone or other full opioid agonists ^19^. Another problem has been that, in spite of the regulatory changes described above, the implementation of buprenorphine treatment has been slow because many waivered physicians are not using it, and there have been difficulties providing the recommended drug counseling and psychosocial services in primary care settings ^20, 21^. The recent approval of a buprenorphine implant (Probuphine) that provides blood levels for 6 months may reduce the diversion problem, but only if it becomes widely used ( http://www.fda.gov/NewsEvents/Newsroom/PressAnnouncements/ucm503719.htm). The involvement of physicians in addiction treatment and the provision of psychosocial services, particularly in U.S. primary care settings, will be dependent on future health care policies that are presently unclear. These problems do not seem to be such important issues in countries with national health care and traditions of parity regarding treatment of substance use and other mental health disorders.

## Extended-release naltrexone

The second newer medication that has been approved by various regulatory bodies is extended-release naltrexone. Naltrexone is an opioid antagonist that blocks the effects of opioids through competitive receptor inhibition and has no opioid agonist effects. A 50 mg tablet has been available since the 1970s that can block opioid effects for 24 hours, and oral doses of 100–150 mg block effects for 48–72 hours. It has a number of advantages, including the absence of tolerance or withdrawal, no risk of diversion, does not magnify the “high” when used with high doses of benzodiazepines or stimulants, does not seem to require dose adjustments when taken with other medications, and has few side effects. Despite these advantages, patients in most western countries have not showed much interest in naltrexone, probably because it has no reinforcing effects and does not attenuate protracted abstinence, and high dropout rates have been the norm.

However, interest was higher in persons under significant legal pressure to stop opioid use ^22^ and in Russia where naltrexone is the only effective relapse prevention medication available and is relatively easy to start because the usual treatment is detoxification and psychosocial treatment and inpatient programs are funded by the national health service and widely available. In this setting, randomized trials have shown that both oral naltrexone and extended-release formulations reduce HIV risk and improve outcomes ^23– 25^, and one study led to the approval of extended-release injectable naltrexone by the U.S. Food and Drug Administration for preventing relapse ^26^.

Australian researchers developed an extended-release implant that blocks opioids for 6 months and has been used with apparent success in several thousand patients ( http://www.staplefordcentre.co.uk/naltrexone-implants.htm); however, it has never been formally approved by a government regulatory body. A variant on the 50 mg naltrexone tablet is nalmefene, also an orally effective antagonist but somewhat longer acting (about 48 hours at dosages of 50–100 mg/day). It has been effective for alcohol treatment ^27^ and shows promise as an alternative to naltrexone for opioid dependence ^28^. These extended-release formulations may be meaningful options for patients who have not done well on agonist treatment or are not interested in it, where other treatment options are unavailable, and in criminal justice settings where individuals are under legal pressure to abstain from opioids– as seen in a recent U.S. study of persons on probation or parole who received extended-release injectable naltrexone ^29^.

## Heroin-assisted treatment

This treatment was developed to help persons who continued regular opioid use while on medically appropriate methadone doses or who would not accept any other treatment. It involves injection or inhalation of heroin two to three times/day in facilities that are medically staffed– so as to quickly respond to overdoses or other adverse interactions. The first studies were done in Switzerland, which showed positive results, and later conducted in the Netherlands, Germany, the United Kingdom, and Canada. Results have been generally positive, and some countries have approved it for this treatment-refractory subgroup of patients ^30^. It is expensive to deliver because of the need for medical staffing with 7 days/week clinic operations that last 10–12 hours/day and, not surprisingly, politically controversial in spite of the positive results from prospective, controlled studies.

## Changes in policy among “traditional” programs

The approval of buprenorphine and extended-release injectable naltrexone has led to more addiction treatment in primary care and criminal justice settings and the addition of medication-assisted treatment to the usual options at well-known substance use disorder treatment programs in the U.S., such as Hazelden and the Caron Foundation ^31^. These programs are national leaders and have relied on psychosocial treatment organized around the 12 steps of Alcoholics Anonymous for the past 50 or more years; thus, these additions have been highly significant within the U.S context ( Maia Szalavitz @maiasz Nov. 05, 2012).

## Psychosocial/behavioral treatments

Most medication-assisted therapy studies have been done in conjunction with psychosocial treatment. Research has called attention to the fact that most patients with opioid and other substance use disorders are ambivalent about stopping ^32^ and that this ambivalence contributes to varying levels of motivation for treatment and abstinence. Miller and Rollnick emphasized that clinicians must be aware of this “normal” ambivalence and developed motivational interviewing and motivational enhancement therapy as ways to resolve this in favor of stopping use ^33^ and moving into “recovery”, recognized as an optimal, though difficult-to-define, outcome ^34^.

## Summary

The development and approval of buprenorphine along with extended-release naltrexone have resulted in more treatment options in more settings with more patients, physicians, and medically trained personnel getting involved in addiction treatment. These changes have occurred at different rates and with varying levels of implementation in different countries and settings and have not been without problems but are advancing. The long-term result is likely to become clearer over the next 10 or more years and will be highly dependent on policy and funding decisions, but available data are encouraging.
